# COVID-19 contact tracing app reviews reveal concerns and motivations around adoption

**DOI:** 10.1371/journal.pone.0273222

**Published:** 2022-09-09

**Authors:** Erica L. Dixon, Sukanya M. Joshi, William Ferrell, Kevin G. Volpp, Raina M. Merchant, Sharath Chandra Guntuku

**Affiliations:** 1 Center for Health Incentives and Behavioral Economics, University of Pennsylvania, Philadelphia, PA, United States of America; 2 Department of Medical Ethics and Health Policy, Perelman School of Medicine, University of Pennsylvania, Philadelphia, PA, United States of America; 3 Department of Computer and Information Science, University of Pennsylvania, Philadelphia, PA, United States of America; 4 Leonard Davis Institute for Health Economics, University of Pennsylvania, Philadelphia, PA, United States of America; 5 Penn Medicine Center for Digital Health, University of Pennsylvania, Philadelphia, PA, United States of America; 6 Department of Emergency Medicine, Perelman School of Medicine, University of Pennsylvania, Philadelphia, PA, United States of America; University of Pisa, ITALY

## Abstract

**Background:**

Google and Apple’s Exposure Notifications System (ENS) was developed early in the COVID-19 pandemic to complement existing contact tracing efforts while protecting user privacy. An analysis by the Associated Press released in December 2020 estimated approximately 1 in 14 people had downloaded apps in states one was available. In this study, we assessed the motivation and experience of individuals who downloaded ENS apps from the Google Play and Apple App Stores.

**Methods:**

We collected review text, star rating, and date of rating for all the reviews on ENS apps in the Google Play and Apple App stores. We extracted the relative frequency of single words and phrases from reviews and created an open vocabulary language, with themes categorized by the research team, to study the salient themes around reviews with high (3–5 stars), neutral (3 stars), and negative (1–2 stars) ratings using logistic regression.

**Results:**

Of 7622 reviews obtained from 26 states between 04/07/2020 to 03/31/2021, 6364 were from Google Play Store, and 1258 were from Apple App Store. We obtained reviews for a total of 38 apps, with 25 apps from the Google Play Store and 13 apps from the Apple Play Store. 78% of the reviews are either 1 star or 5 stars. Positive reviews were driven by ease of use, support for the state government in creating the app, and encouragement for others to download, as well as engage in other COVID-19 precautions. Negative and neutral reviews focused on issues with app functionality (i.e., installation and tracking errors).

**Conclusions:**

Uptake was the largest barrier to success for ENS apps, but states can use insight from app store reviews to better position themselves if they choose to develop further public health apps.

## Introduction

Early in the COVID-19 pandemic, Google and Apple collaborated to launch an Exposure Notification System (ENS), which utilized an Application Programming Interface (API) allowing smartphones to share encrypted, anonymous keys between devices using Bluetooth technology [1, 2]. The API was structured so keys would be exchanged when phones, each with the app installed, were in close contact; the goal was to be able to provide exposure notifications if a close contact later tested positive for COVID-19. This was meant both to alleviate the pressure on traditional contact tracing efforts, as well as counteract situations traditional contact tracing cannot work, i.e., when contacts are unknown to a case.

This API was provided to state departments of health, who could then develop apps for the citizens of their state. Individual states determined what their apps looked like and what functions they included, as well as the parameters for what was considered a close contact (e.g., 15 minutes of exposure at 6 feet or less). Over the course of the pandemic, several states launched Exposure Notification Apps, with the vast majority engaging with the ENS technology created by Google and Apple. The uptake of these apps has remained low throughout the COVID-19 pandemic [3–5]; while numbers of downloads are not typically publicly available, an analysis by the Associated Press released in December 2020 estimated approximately 1 in 14 people had downloaded apps in states an app was available.

Much research and commentary have focused on why people would not download these apps or why they would not be useful, citing privacy concerns and likely low uptake, as well as higher download rates by risk-averse populations already engaging in protective action [6–8]. Efforts to increase downloads such as informational campaigns appear to have little impact; while financial incentives appear to produce a large increase in downloads in study populations, states have not provided this as a path to increase downloads [9, 10].

While downloads of the app are likely to remain a large barrier, another challenge is the successful maintenance of the population who have downloaded COVID tracking apps. The focus of this study is on the motivation and experience of people who downloaded ENS apps, with data taken from reviews for each of the individual state apps. Several works have studied app reviews in the past to identify user experiences along with bug reports and feature requests for different mobile apps [11]. An analysis of European contract tracing apps [12], found predominantly negative reviews, suggesting issues with battery life and a lack of notifications which motivated the negative scores. Table 1 summarizes prior works studying mobile app reviews.

**Table 1:** Summary of prior works studying mobile app reviews.

Study	Topic of Interest	App category	# Reviews	# Apps
Aslam et al. [23]	Classifying App Review Types	Productivity, Travel, Social, Photography, Communication	1,272,510	Google: 80, Apple: 1100
Guo et al. [24]	Characterizing User Issues in App Reviews	All Genres (Books, Business, Entertainment, Productivity, Social, etc)	5,867,198	Apple: 151
Guzman et al. [25]	Sentiment Analysis of Reviews	Games, Productivity, Travel, Photography, Social, Communication	32,210	Google: 3, Apple: 7
Maalej et al. [26]	Classifying App Review Types	Productivity, Travel, Social, Photography, Communication	1,303,182	Google: 40, Apple: 1100
Martens et al. [27]	Detecting Fake App Reviews	All Genres (Books, Business, Entertainment, Productivity, Social, etc)	62,625,644	Apple: 1,432,020
Maalej et al. [28]	Classifying App Review Types	Productivity, Travel, Social, Photography, Communication	1,272,510	Google: 80, Apple: 1100
McIlroy et al. [29]	Characterizing User Issues in App Reviews	Entertainment, Productivity, Social, Games, Sports, Shopping, Books	230,277	Google: 20, Apple: 4
Panichella et al. [30]	Classifying App Review Types	Games, Travel, Social, Productivity, Photography	32,210	Google: 4, Apple: 3

We undertake a computational study of the reviews given to COVID-19 contact tracing apps in the United States, where we categorize reviews into positive, neutral, and negative categories in order to better understand user motivations and experience with the apps. App reviews are utilized to provide an important overview of motivations for download, direct experience of the apps, and feedback on improvements. Insights from the analysis using natural language processing [13] of these reviews can contribute to understanding how to maintain the population of active app users.

## Methods

### Data collection

We identified the official contact tracing apps released by different states in the United States and obtained the app links on Google Play and Apple App Stores. For each app, we web-scraped the review text, star rating, and date of rating using a python script (packages: google-play-scraper and apple-store-scraper). In Table 2, we have listed a breakdown of the number of reviews per state and platform. Note that North Dakota (ND), South Dakota (SD), and Wyoming (WY) used the same ENS App. This was approved as an exempt study by the University of Pennsylvania Institutional Review Board. The collection and analysis of the data complied with the terms of service for the source of the data.

**Table 2:** Number of COVID-19 contact tracing app reviews per platform and per state.

Platform	# of reviews
Google	6364
Apple	1258
Total	7622
**State**	**# of reviews**
UT	1726
VA	677
NY	609
PA	555
NC	428
CA	401
ND/SD/WY	363
NJ	356
MI	330
MN	314
CO	295
WA	261
RI	254
NV	249
MD	162
AL	154
CT	115
HI	72
WI	69
DE	55
LA	54
AZ	51
NM	38
DC	34

### Data preprocessing

We created a column to categorize positive, neutral, and negative reviews. 4–5 star ratings were labeled as positive, 3-star ratings were labeled as neutral, and 1–2 star ratings were labeled as negative. In order to process the app reviews, we used the HappierFunTokenizer available with the DLATK package [14]. We represented the language of each app review as a set of features. We labeled the top 10 most frequent words in the review text as stopwords and removed them from our dataset. We then extracted 1-,2-, and 3-grams from all app reviews to analyze significant associations between words & phrases and positive, neutral, and negative themes.

### Language feature extraction

We extracted the relative frequency of single words and phrases from reviews and created an open vocabulary language feature set using Latent Dirichlet Allocation (LDA) [15]. LDA uses an unsupervised clustering algorithm to identify latent topics in large quantities of text. The topics are generated from the data and are clusters of words in the closed-vocabulary approach. The algorithm assumes that each word occurrence can be attributed to one or more topics generated from the corpus. Words are assigned to a topic based on co-occurrence with other words across the corpus of user reviews and repeated until all of the words are designated to a set of topics with other semantically similar words. These topics represent semantically coherent clusters of words in which words are assigned weights based on their likelihood of occurring within each topic. The number of topics is assigned a priori, and for this study, we obtained 25 data-driven topics using LDA, as well as other topic modeling algorithms such as Contextualized Topic Modeling (CTM) and Non-Negative Matrix Factorization (NMF). The top 20 words per topic using LDA, CTM, and NMF are listed in the Supplementary document. After calculating each of their topics’ coherence score, a measure used to assess the quality of topics, we found that LDA had the highest values (see S2 Table for scores). As a result, we decided to use LDA as our topic modeling technique to understand user reviews. The distribution of LDA topics was extracted for positive, negative, and neutral reviews, and themes were categorized by an independent review by the research team.

### Statistical analyses

We categorized 4 and 5-star ratings as positive, 3-star ratings as neutral, and 1–2 star ratings as negative. Logistic regression was used to identify topics associated with review ratings [14]. The effect size was measured using odds ratio. We extracted 10 reviews with the highest topic prevalence for each positive, neutral, and negative topic; two independent reviewers assigned themes to topics with a third independent reviewer adjudicating any differences. We used Benjamini-Hochberg p-correction and *p<0*.*05* for indicating meaningful associations.

## Results

Of 7622 reviews obtained from 26 states between 04/07/2020 to 03/31/2021, 6364 were from Google Play Store and 1258 were from Apple App Store. We obtained reviews for a total of 38 apps, with 25 apps from the Google Play Store and 13 apps from the Apple Play Store. Consistent with prior works studying user reviews on online platforms [16–18], the distribution of ratings follows a bimodal distribution as shown in S1 Fig. ~78% of the reviews are either 1 star or 5 stars. This follows a known trend in product reviews where users who had more salient experiences, either negative or positive, are more motivated to leave reviews than others with less salient experiences [19], suggesting that users who had a relatively middle-of-the-road experience did not comment as much on the app store reviews (~22% of the reviews had a 2–4 star rating).

**Fig 1:**
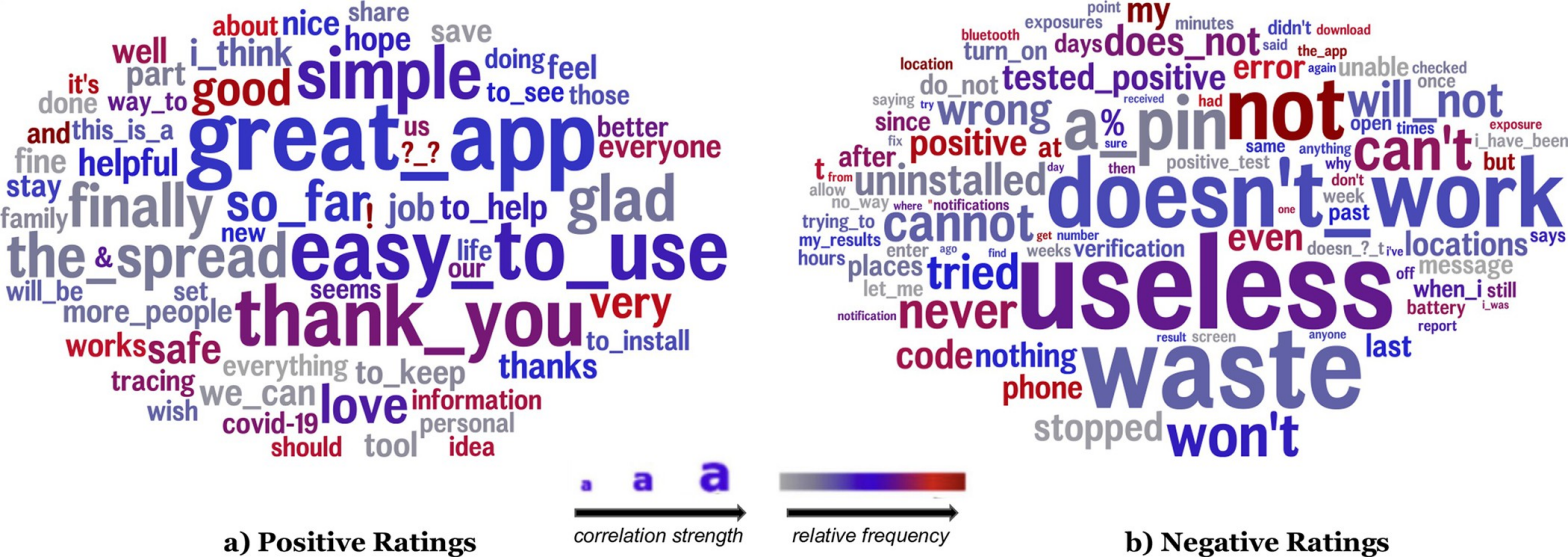
Words and phrases associated with positive reviews (a) and negative reviews (b). Only one word (‘but’) was significantly associated with neutral ratings. Word size indicates the strength of correlation and word color indicates relative word frequency (p<0.05, Benjamini-Hochberg p-corrected).

Words and phrases (Fig 1) associated with positive reviews are consistent with the themes in LDA topics: positive reviews consist of ease of use (‘easy to use’, ‘great app’, ‘simple’), encouragement for others to also install the app (‘everyone’, ‘share’, ‘helpful’, ‘the spread’), and gratitude (‘thank you’, ‘glad’). Negative reviews are dominated by complaints that the app is not useful (‘waste’, ‘useless’) and has inaccuracies and functional issues (‘cannot’, ‘doesn’t work’, ‘stopped’, ‘wrong’). For neutral reviews, there was only one word associated with this category (‘but’).

**Fig 2:**
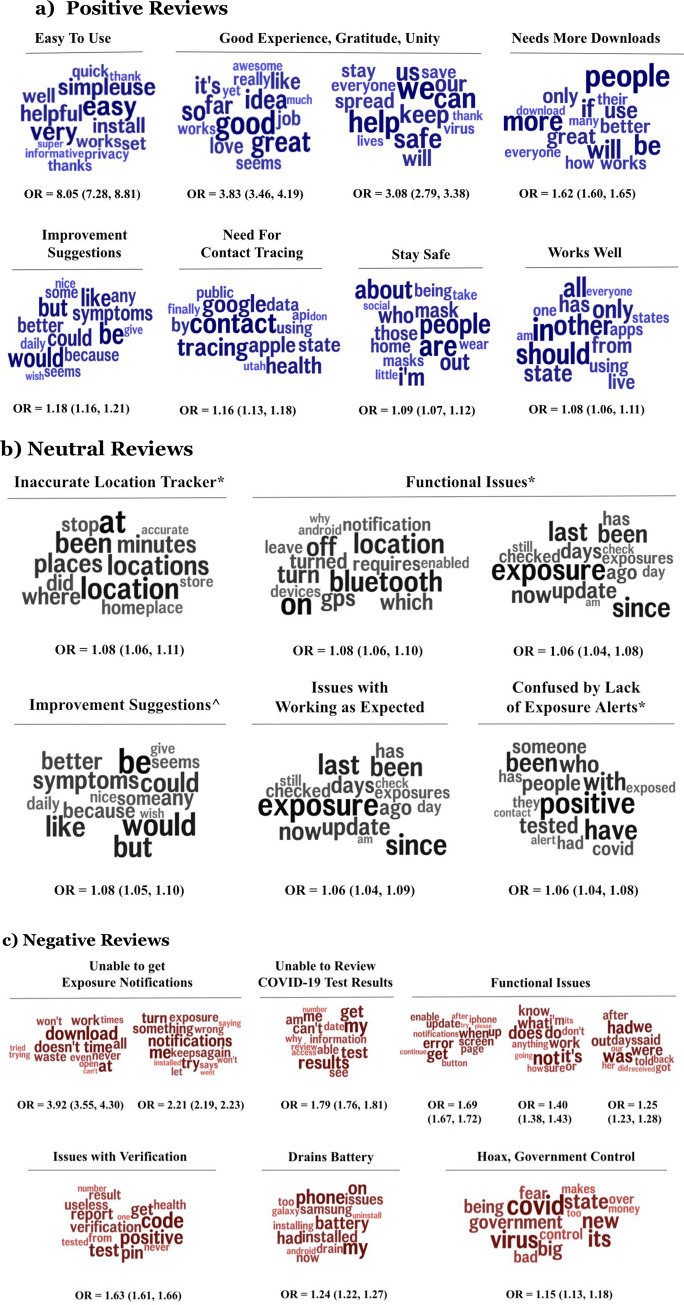
Topic word clouds associated with positive (in blue), neutral (in black), and negative (in red) reviews. Topics related to positive reviews correspond to 4–5 star ratings. Word size indicates the strength of correlation and word color indicates relative word frequency (p<0.05, Benjamini-Hochberg p-corrected). Odds ratios (OR) and confidence levels are listed below the word clouds. Note: The topics in neutral reviews indicated with a * overlap with negative reviews, and the topic indicated with ^ overlaps with positive reviews.

Themes significantly associated with positive, neutral, and negative reviews are shown in Fig 2 along with an odds ratio that represents the odds that a topic will occur in one group of reviews (categorized by their ratings), compared to the other group. In our case, the three groups are a) positive reviews with 4–5 star ratings, b) neutral reviews with 3-star ratings, and c) negative reviews with 1–2 star ratings. Out of the 7622 reviews, 3537 were positive, 571 were neutral, and 3514 were negative. A full list of all topics along with example reviews can be found in S1 Table.

Positive reviews consist of the app being user-friendly (‘easy’, ‘simple’, ‘helpful’, ‘quick’, ‘informative’, Odds-ratio, OR = 8.05), having sentiments of encouragement and gratitude (‘good’, ‘great’, ‘idea’, ‘job’, ‘love’, OR = 3.83; ‘we’, ‘can’, ‘help’, ‘safe’, stop’, ‘spread’, OR = 3.08), urging others to download (‘people’, ‘more’, ‘be’, ‘will’, ‘if’, ‘use’, ‘great’, OR = 1.62), suggestions for improvement (‘could’, ‘give’, ‘daily’, ‘symptoms’, ‘better’, OR = 1.18), need for contact tracing (‘contact’, ‘tracing’, ‘google’, ‘health’, ‘apple’, ‘state’, ‘data’, OR = 1.16), staying safe (‘are’, ‘people’, ‘about’, ‘home’, ‘wear’, ‘masks’, OR = 1.09), and that the app works well (‘in’, ‘should’, ‘other’, ‘state’, ‘using’, ‘apps’, ‘everyone’, OR = 1.08).

Neutral reviews mainly consist of negative themes. Three out of the five themes overlapped with negative themes, specifically perceived inaccuracies in tracking location (these apps do not track individual location, but these are reflective of user perception) (‘locations’, ‘places’, ‘minutes’, ‘where’, ‘stop’, ‘home’, ‘accurate’, OR = 1.08), functional issues (‘on’, ‘bluetooth’, ‘location’, ‘off, ‘gps’, ‘requires’, ‘notification,’ ‘why,’ OR = 1.08; ‘exposure’, ‘since’, ‘last’, ‘been’, ‘now’, ‘update’, ‘days’, ‘ago’, ‘check’, OR = 1.06), and confusion by the lack of exposure alerts (‘positive’, ‘tested’, ‘people’, ‘someone’, ‘covid’, ‘exposed’, ‘alert’, ‘contact’, OR = 1.06). The other themes corresponded to suggestions for improvement (‘could’, ‘give’, ‘daily’, ‘symptoms’, ‘better’, OR = 1.08) which overlapped with the positive theme, as well as issues with working as expected (‘way’, ‘find’, ‘please’, ‘needs’, ‘issue’, ‘user’, ‘option’, ‘made’, OR = 1.06).

Furthermore, themes associated with negative reviews consist of complaints about not receiving exposure notifications (‘doesn’t’, ‘all’, ‘work’, ‘waste’, ‘never’, ‘won’t’, ‘open’, OR = 3.92; ‘me’, ‘notifications’, ‘try’, ‘turn’, ‘something’, ‘again’, ‘exposure’, OR = 2.21), inaccuracy in tracking location (‘locations’, ‘places’, ‘minutes’, ‘where’, ‘stop’, ‘home’, ‘accurate’, OR = 1.83), not being able to access testing results (‘my’, ‘results’, ‘get’, ‘test’, ‘me’, ‘can’t’, ‘see’, ‘information’, ‘why’, OR = 1.79), functional issues (‘get’, ‘error’, ‘screen’, ‘notifications’, ‘continue’, ‘try’, ‘please’, OR = 1.69; ‘exposure’, ‘since’, ‘last’, ‘been’, ‘now’, ‘update’, ‘days’, ‘ago’, OR = 1.66; ‘not’, ‘it’s’, ‘does’,‘what’, ‘work’, ‘know’,‘don’t’, ‘anything’, ‘how’, ‘going’, OR = 1.40; ‘was’, ‘had’, ‘were’, ‘out’, ‘said’, ‘days’, ‘after’, ‘told’, ‘got’, ‘back’, ‘received’, OR = 1.25), issues with verification (‘code’, ‘positive’, ‘test’, ‘pin’, ‘get’, ‘report’, ‘verification’, ‘result’, ‘useless’, ‘health’, ‘from’, ‘never’, ‘tested’, OR = 1.63), how the app drains battery (‘my’, ‘phone’, ‘battery’, ‘had’, ‘installed’, ‘issues’, ‘samsung’, ‘drain’, ‘installing’, ‘galaxy’, ‘android’, ‘uninstall’, OR = 1.24), and concerns of government control (‘covid’, ‘government’, ‘bad’, ‘fear’, ‘control’, ‘makes’, ‘money’, OR = 1.15).

## Discussion

Reviews of COVID-19 contact tracing apps reveal motivations around the adoption of these apps and provide reflections on user experience post-download. These reviews can be viewed as serving multiple purposes—first, providing important feedback to the app developers and state governments on user experience, and second, functioning as either positive or negative marketing for potential new users.

Positive reviews were driven by a combination of ease of use of the app, and encouragement for others to download the app, as well as engage in other COVID-19 precautions (i.e. staying safe, wearing masks). Negative reviews primarily focused on issues with app functionality, such as installation errors, battery drainage, inaccuracies in tracking location (notably, these apps do not track the location of the user, but some users may perceive that they do), and perceptions that the app was useless as people were not receiving notifications about exposures. Neutral reviews (2–4 on the rating scale) consisted of predominantly negative feedback, including inaccuracies in tracking location, functional issues, and confusion regarding the lack of exposure alerts. There were also suggestions for improvement and reflections on how people had issues with the app working as they would have liked to, suggesting positive feelings about using the app, if functioning correctly. As ~22% of reviews were non-extreme reviews (2–4 star ratings), most people who left an app review either felt positively or negatively about the app. To analyze further, since ~78% of the app reviews were either categorized as a 1-star rating or a 5-star rating, accounting for the U-shape distribution in S1 Fig, the individuals who were most likely to leave a review were the ones who exhibited extreme sentiments (either very positive or negative) towards the app. Individuals who had non-extreme sentiments towards the app were less likely to comment.

A clear theme that emerged from reviews is that once people download, the experience needs to be a good one; apps must be easy to install, easy to use, and easy to understand. Negative and neutral reviews reflected numerous functional issues, which could be addressed by clearer explanations either from state communications campaigns or from in-app messaging and explanations.

Notably, positive, negative, and neutral reviews all reflect the primary issue with these apps–that not enough people have downloaded them within the state for them to be truly successful in providing meaningful notifications of encounters. The app can only provide notifications of exposures if people who test positive have downloaded the app and agreed to anonymously share their positive status. Positive reviews frame this as a problem of more people needing to download the app (i.e. everyone should download), as well as states needing to do more marketing for the apps, while negative reviews focus on not receiving notifications and the app itself not being useful (i.e. not receiving exposure notifications).

Privacy was not a frequently discussed concern in the app reviews, which may reflect to some degree that those potential users most concerned about privacy did not download the app. However, it does suggest that, at least for users leaving reviews, the experience of downloading and using the app did not raise privacy concerns. Prior works also did not find privacy to be a major concern but observed that themes prioritizing user-friendliness and interface while studying diabetes self-management [20], finance apps [21], mental health apps [22], were significant for user uptake.

### Limitations

This study has several limitations. First, themes could vary by platform (Google vs Apple) and by state but the sample size was not powered to compare insights by platform. Second, the thematic annotations for topics associated with high, neutral, and negative ratings could suffer from bias. Third, some states chose to adopt the Exposure Notification Express service from Apple, which functions through the settings menu on an iPhone and does not have an associated app (CO, WA, NM, etc.). States that selected this option only have Google reviews. Finally, the individuals downloading and reviewing the apps may not be representative of the population in the states, or even of the population who downloaded the apps, as people with stronger experiences (positive and negative) may have been more likely to leave a review.

Since only 1 in 14 people had downloaded Exposure Notification Apps (ENS) apps in states this app was available [3], it is reasonable to conclude that one of the largest barriers to success for ENS apps was uptake; these apps largely failed to become widely used in the United States despite significant initial optimism that they would be an important tool in combating COVID spread. If getting people to download ENS apps is the biggest challenge, prioritizing a positive user experience so numbers aren’t lost post-download could be a priority for states utilizing these apps. Nonetheless, by learning from the successes and failures of the app for people who chose to download it, states can position themselves to have more successful public health apps in the future. App Store reviews are a good source of insight for states on user experience, both positive and negative.

## Supporting information

S1 Fig Distribution of ratings by platform. (DOCX)

S1 Table Topics associated with positive, neutral, and negative reviews. Only significant topics after Benjamini-Hochberg p-correction (p<0.05) are shown. Topics sorted by Odds Ratio are shown in this table.
(DOCX)

S2 Table Comparison of coherence scores using Latent Dirichlet Association (LDA), Contextualized Topic Modeling (CTM), Non-Negative Matrix Factorization (NMF). Calculated the coherence scores using four coherence measures: u_mass (-14,14), c_v (0, 1), c_uci (0,1), and c_npmi (0,1).
(DOCX)
