# Supplementary figures and images for: COVID-19 contact tracing app reviews reveal concerns and motivations around adoption
Source: PLoS One. 2022 Sep 9;17(9):e0273222. doi: 10.1371/journal.pone.0273222 (PMC9462778; doi:10.1371/journal.pone.0273222)

**Supplementary Figure S1: Distribution of ratings by platform**

**
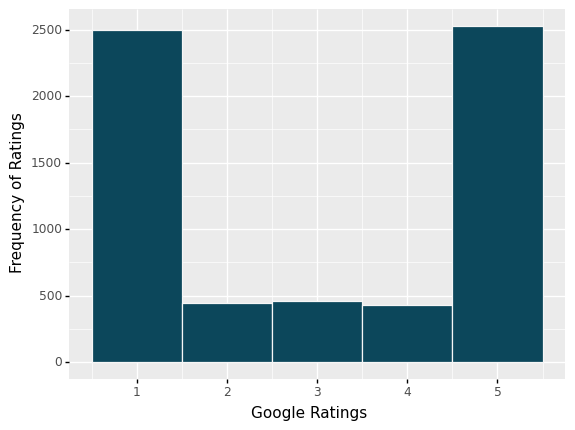

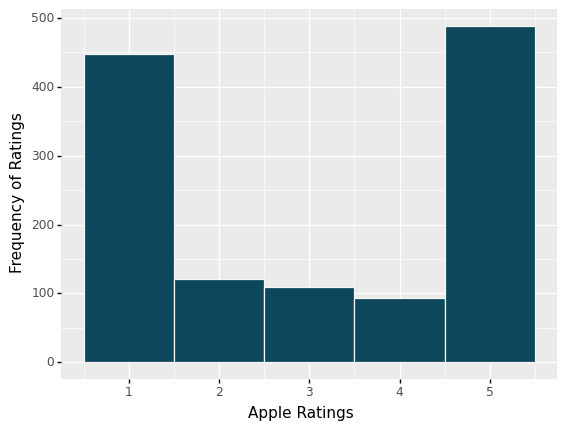
**

Supplement: S1 Fig — (DOCX) [file pone.0273222.s001.docx]
