# Supplementary material for: COVID-19 contact tracing app reviews reveal concerns and motivations around adoption
Source: PLoS One. 2022 Sep 9;17(9):e0273222. doi: 10.1371/journal.pone.0273222 (PMC9462778; doi:10.1371/journal.pone.0273222)
Supplement: S1 Table — Only significant topics after Benjamini-Hochberg p-correction (p<0.05) are shown. Topics sorted by Odds Ratio are shown in this table. (DOCX) [file pone.0273222.s002.docx]

**Supplementary Table S1. Topics associated with positive, neutral, and negative reviews.** Only significant topics after Benjamini-Hochberg p-correction (p<0.05) are shown. Topics sorted by Odds Ratio are shown in this table.

| **High Rating (4-5 stars)** | | | |
| --- | --- | --- | --- |
| **Topic Theme** | **Top Words** | **Odds Ratio (95% CI)** | **Example Reviews** |
| Easy To Use | easy, very, use, simple, helpful, install, well, set, works, quick, thanks, privacy, thank, informative, super | 8.05 (7.28, 8.81) | "Easy to navigate"  "Easy to install and very informative" |
| Good Experience, Gratitude, Unity | good, great, so, idea, far, it's, like, love, job, seems, really, works, yet, much, awesome | 3.83 (3.46, 4.19) | "Good so far"  "great excellent service"  "Awesome idea!" |
|  | we, help, can, safe, us, keep, our, will, spread, stay, save, everyone, virus, lives, thank | 3.08 (2.79, 3.38) | "Thank you CA! Proud to download and help save lives."  "Stay Safe MN!"  "Let's solve the pandemic together." |
| Needs More Downloads | people, more, be, will, if, use, great, only, better, works, how, their, everyone, many, download | 1.62 (1.60, 1.65) | "At 10k downloads, roughly 0.71% of Hawaii's 1,416,000 population are using this app...It's a great app, but more people need to know about it to be truly useful." |
| Improvement suggestions | be, would, but, like, could, symptoms, better, any, because, some, seems, give, nice, daily, wish | 1.18 (1.16, 1.21) | "Wish it would remind me to enter daily data. And with 35,000 users and only 180 check-ins a day I assume that feature would help others too." |
| Need For Contact Tracing | contact, tracing, google, health, apple, state, by, data, using, public, api, don, finally, utah | 1.16 (1.13, 1.18) | "We are finally getting an official contract tracing app! Everyone download please"  “This application will significantly improve our ability to track this virus, perform contact tracing, and prevent major outbreaks.” |
| Stay Safe | are, people, about, i'm, out, who, mask, those, home, being, wear, masks, little, take, social | 1.09 (1.07, 1.12) | "I hope everyone uses this app. Wear a mask, stay safe, practice social distance, and vaccinate." |
| Works Well | in, should, other, all, only, state, has, from, live, using, apps, one, states, am, everyone | 1.08 (1.06, 1.11) | "Good and perfect"  "Fast and easy"  "I don't live in Nevada I live in Michigan" |
| Inaccurate Numbers | about, cases, how, information, county, users, also, some, area, data, see, numbers, could, many, where | 1.06 (1.04, 1.08) | "Lacking in functionality to inform users... Doesn't link outside the app to get additional up-to-date information and directives from government agencies." |
| **Neutral Rating (3 stars)** | | | |
| Location Tracker Is Inaccurate | at, location, been, locations, places, minutes, where, did, stop, home, store, place, 10, accurate | 1.08 (1.06, 1.11) | "Locations are not always accurate. Shows me stopping alone a route when I did not stop. Sometimes for minutes, sometimes hours...." |
| Functional Issues | on, bluetooth, location, off, turn, gps, which, turned, requires, notification, leave, devices, enabled, android, why | 1.08 (1.06, 1.10) | "Drains battery due to bluetooth usage...."  "Had to uninstall to fix bluetooth connection problems with Fitbit and car audio bluetooth" |
|  | exposure, since, last, been, now, update, days, ago, has, checked, exposures, day, still, check, am | 1.06 (1.04, 1.08) | "The app hasn't checked my exposure since dec 24th. Its now jan 24th. Seems pretty useless."  "The app isn't actually checking for exposure. Opened it today and it was last checked for exposure three weeks ago." |
| Improvement suggestions | be, would, but, like, could, symptoms, better, any, because, some, seems, give, nice, daily, wish | 1.08 (1.05, 1.10) | "Wish it would remind me to enter daily data. And with 35,000 users and only 180 check-ins a day I assume that feature would help others too." |
| Issues With Working as Expected | as, no, an, there, well, out, way, find, please, needs, issue, also, user, option, made | 1.06 (1.04, 1.09) | “Please consider a patch for phones running older versions of iOS! Not everyone has the newest fanciest technology and it limits this apps reach!”  “Important and necessary.”  “Works as described and safe.” |
| Confused By (Lack Of) Exposure Alerts | positive, have, been, with, who, tested, people, someone, covid, has, had, they, exposed, alert, contact | 1.06 (1.04, 1.08) | "Lacking in functionality to inform users... Doesn't link outside the app to get additional up-to-date information and directives from government agencies." |
| **Low Rating (1-2 stars)** | | | |
| Unable To Get Exposure Notifications | download, at, time, doesn't, all, work, waste, never, won't, even, times, open, trying, tried, can't | 3.92 (3.55, 4.30) | "...since December 16, it has been inactive. I have restarted the phone, checked and updated settings, still it shows Dec 16. I was really excited about this app only to be disappointed after a few months." |
|  | me, notifications, try, turn, something, again, exposure, keeps, wrong, let, says, won't, saying, installed, went | 2.21 (2.19, 2.23) | "Cannot turn on exposure notifications"  "Whenever I try to turn on exposure notifications it always says something is wrong." |
| Location Tracker Is Inaccurate | at, location, been, locations, places, minutes, where, did, stop, home, store, place, 10, accurate | 1.83 (1.81, 1.86) | "Locations are not always accurate. Shows me stopping alone a route when I did not stop. Sometimes for minutes, sometimes hours...." |
| Unable To Review COVID-19 Test Results | my, results, get, test, me, can't, am, see, able, information, date, why, review, access, number | 1.79 (1.76, 1.81) | "my test results are now blocked because my date of birth was input incorrectly"  "Can't get past confirm on birth date and age it's locked up"  "App sux. Can't even review the results" |
| Functional Issues | get, error, when, up, screen, update, page, enable, notifications, button, iphone, after, continue, try, please | 1.69 (1.67, 1.72) | "Getting error code 1002 when trying to open"  "Can't get past the third page of setup" |
|  | exposure, since, last, been, now, update, days, ago, has, checked, exposures, day, still, check, am | 1.66 (1.63, 1.68) | "The app hasn't checked my exposure since dec 24th. Its now jan 24th. Seems pretty useless."  "The app isn't actually checking for exposure. Opened it today and it was last checked for exposure three weeks ago." |
|  | on, bluetooth, location, off, turn, gps, which, turned, requires, notification, leave, devices, enabled, android, why | 1.43 (1.41, 1.46) | "Drains battery due to bluetooth usage...."  "Had to uninstall to fix bluetooth connection problems with Fitbit and car audio bluetooth" |
|  | not, it's, does, do, what, work, know, or, i'm, sure, don't, anything, how, its, going | 1.40 (1.38, 1.43) | "App does not function properly"  "Not sure it works"  "Useless Doesn't do anything." |
|  | was, had, we, were, out, said, days, after, told, got, back, our, her, did, received | 1.25 (1.23, 1.28) | "This app is junk...My wife is Covid19 positive. She reported in the app as infected patient. I stood right next to her (with her phone on her hand)and the app on my phone did not alert anything..." |
| Issues with verification | code, positive, test, pin, get, report, verification, result, useless, health, from, never, tested, number, one | 1.63 (1.61, 1.66) | “Unable to enter pin”  “Tested positive, can't find verification code” |
| Confused By (Lack Of) Exposure Alerts | positive, have, been, with, who, tested, people, someone, covid, has, had, they, exposed, alert, contact | 1.42 (1.39, 1.44) | "I have been around multiple people whom have tested positive...have gotten zero notifications. Not very effective."  "I know I've been potentially exposed and have received no exposure notifications" |
| Drains Battery | my, phone, on, battery, had, installed, issues, samsung, now, drain, too, installing, galaxy, android, uninstall | 1.24 (1.22, 1.27) | "Drains battery due to bluetooth usage...."  "The app was killing my battery too fast. I had to uninstall. I'm using a Samsung Galaxy S9." |
| Hoax, Government Control | covid, virus, its, new, state, big, government, being, bad, fear, over, control, makes, money, too | 1.15 (1.13, 1.18) | "Government tracking at its finest. This is absolute garbage just like Governor Cooper"  "This is like Communist China."  "Its a hoax" |
