# Supplementary material for: COVID-19 contact tracing app reviews reveal concerns and motivations around adoption
Source: PLoS One. 2022 Sep 9;17(9):e0273222. doi: 10.1371/journal.pone.0273222 (PMC9462778; doi:10.1371/journal.pone.0273222)
Supplement: S2 Table — Calculated the coherence scores using four coherence measures: u_mass (-14,14), c_v (0, 1), c_uci (0,1), and c_npmi (0,1). (DOCX) [file pone.0273222.s003.docx]

**Supplementary Table S2: Comparison of Coherence Scores using Latent Dirichlet Association (LDA), Contextualized Topic Modeling (CTM), Non-Negative Matrix Factorization (NMF).** Calculated the coherence scores using four coherence measures: u_mass (-14,14), c_v (0, 1), c_uci (0,1), and c_npmi (0,1).

| **Coherence Scores using LDA, CTM, and NMF** | | | |
| --- | --- | --- | --- |
| Coherence Measure | LDA | CTM | NMF |
| u_mass | -6.5182 | -7.8960 | -8.5439 |
| c_v | 0.3746 | 0.2049 | 0.2316 |
| c_uci | -0.9500 | -2.4325 | -3.4348 |
| c_npmi | -0.0009 | -0.0976 | -0.1289 |
